# Exploring Zentangle as a virtual mindfulness-based art intervention for people with serious mental illness

**DOI:** 10.3389/fpsyt.2023.1260937

**Published:** 2023-11-30

**Authors:** Marko Stojcevski, Amy Cheung, Victor Agwu, Xiaoduo Fan

**Affiliations:** Department of Psychiatry, University of Massachusetts Chan Medical School/UMass Memorial Health, Worcester, MA, United States

**Keywords:** serious mental illness (SMI), mindfulness, creative arts, mindfulness-based art intervention, art therapy, Zentangle

## Abstract

**Introduction:**

Zentangle is an emerging art intervention that incorporates mindfulness into creative drawing. This pilot study explored Zentangle as a novel adjunct treatment for people with serious mental illness (SMI).

**Methods:**

Six participants with SMI completed an 8-week Zentangle program. Psychiatric outcomes were evaluated using the Brief Psychiatric Rating Scale (BPRS), Mindful Attention Awareness Scale (MAAS), Perceived Stress Scale (PSS), and Quality of Life Enjoyment and Satisfaction Scale (Q-LES-Q-SF). A focus group was conducted to better understand the experiences of the participants.

**Results:**

A significant reduction in psychiatric symptoms was observed as measured by the total score on the BPRS between baseline and 5-week post-intervention (40.7 ± 9.1 vs. 33.7 ± 8.9, mean ± SD, *p* = 0.02). Participants also showed a significant increase in mindful attention using the average score on the MAAS between 1- and 5-week post-intervention (3.5 ± 0.4 vs. 4.2 ± 0.7, mean ± SD, *p* = 0.04). Four themes were generated from the focus group: (1) approaching mindfulness through Zentangle; (2) power of uncomplicated art creation; (3) understanding the value of self-appreciation; and (4) fostering a positive environment.

**Discussion:**

Our preliminary data suggest that the use of Zentangle for participants with SMI may have a positive impact on overall psychiatric symptoms and mindfulness. Moreover, the Zentangle Method encourages positive emotions like gratitude and self-accomplishment to counteract negative feelings of self-criticism and failure in participants.

## 1 Introduction

Serious mental illness (SMI) constitutes a specific category within the spectrum of mental health conditions and is characterized by severe and debilitating symptoms that can lead to functional impairment and limit activities of daily living. It commonly refers to a diagnosis of schizophrenia, bipolar disorder, or major depressive disorder (1). Individuals with SMI face considerable health disparities with mortality rates that are reported to be 2–3 times higher than that of the general population (2, 3). Conventional treatment approaches for SMI include medication treatment and psychotherapy. Yet, currently available medications have significant limitations due to neurological and metabolic side effects (4). Previous studies have found that 10–30% of people diagnosed with schizophrenia are treatment-resistant (5, 6). These challenges have led to the exploration of complementary approaches including creative arts interventions to treat individuals with SMI.

Creative arts interventions (7) are a multi-method artistic approach broadly encompassing visual arts, musical engagement, movement and dance, drama and theater, and creative writing (8, 9). Several modalities of creative arts have been studied in the SMI population (10) and reports on visual arts programs for people with psychotic disorders are emerging (11, 12). Interventions focused on visual arts have been shown to improve one's cognitive and/or psychological wellbeing by allowing participants to express themselves creatively through visual tasks such as drawing, painting, collage, clay modeling, and sculpting. The creation of visual objects or experiences can either be directed or undirected and foster individual creativity, emotional expression, and memories (13). Chu et al. demonstrated that visual arts programs using calligraphy can significantly enhance cognitive function and improve symptoms of anxiety, depression, and psychosis (14). Visual arts interventions performed in a group setting were also found to increase overall wellbeing by providing a supportive environment for people with SMI to express feelings of hope and relieve negative feelings (15). As visual arts programs entail various approaches and complex practices, more research is needed to better understand specific methods of implementation in this patient population.

Mindfulness has been defined as “*paying attention in a particular way: on purpose, in the present moment, and non-judgmentally*” (16). In contrast to mind-wandering states, the ability to focus on the current moment has been linked to improved wellbeing (17). Self-reported levels of mindfulness among people with schizophrenia have been found to be lower when compared with healthy controls, raising the possibility that maladaptive behaviors caused by psychiatric illness can be attributed to mind-wandering states (18). This idea has led to the exploration of mindfulness interventions and their effectiveness in improving psychotic symptoms among those living with SMI (19). Mindfulness-based interventions have been shown to increase emotional regulation and improve negative symptoms in people with SMI (20, 21). Chadwick et al. incorporated the notion of mindfulness into treatment tailored to the needs of people living with SMI and showed an overall clinical improvement among those participating in mindfulness-based interventions (22).

In recent years, there has been a growing interest in mindfulness-based art interventions within the mental health care field. The structure of these programs is focused on implementing mindful teachings fused with diverse forms of creative arts. Most research on mindfulness-based art interventions has focused on populations with cancer, suggesting that it may be effective in reducing stress and improving quality of life (23–25). Studies using mindfulness-based art interventions within the SMI population remain sparse.

Zentangle is a guided mindfulness-based art intervention that strives to promote individual confidence and appreciation of ability and encourages non-judgmental improvement in self-image and analytical skills (26). Structured in a group format, participants engage in the spontaneous creation of images and patterns on a paper tile to channel creativity through drawing (27, 28). Each unique art piece is shared and then combined into a mosaic highlighting individual and collective interpretations of the art. This social aspect of art creation has been shown to combat feelings of alienation, helplessness, and emotional deprivation (29). Zentangle relies on a non-verbal expression that allows participation from individuals who struggle with communication including those who are illiterate or mute (30). Conversely, Zentangle enriched linguistic experiences in children aged 3–5 years by embracing and adapting to cultural and language diversity (31). A pilot study analyzing Zentangle demonstrated improved cognition in older adults with mild cognitive impairments, suggesting the potential benefit of Zentangle in reducing the risk of dementia and promoting healthy aging (32). Using the waitlist-controlled method, Chan and Lo were able to demonstrate significant improvements in depressive symptoms and self-compassion among older adults with depression who participated in the Zentangle group. The improvements were noted as both immediate and maintenance effects at the 6-week follow-up (33).

Important community-based facilities utilized by individuals with SMI for psychosocial support, such as community health centers and day programs, have been closed during the COVID-19 pandemic. Innovative care delivery methods to support individuals with SMI were developed during this time of social distancing. The study evaluating the mixed-mode (face-to-face and online) intervention of Pastel Nagomi Art and Zentangle Art showed positive intervention effects, as both intervention groups improved in regard to depression, and positive affect (34). Many clinical services and community resources have shifted their operations to virtual platforms so that individuals with SMI can continue to receive medication treatment and psychosocial support (35). Support for accessibility and feasibility of telehealth in patients with SMI was described prior to the pandemic even though it was not widely utilized (36). The pandemic opened an opportunity to re-evaluate the role of telehealth in this patient population. Miu et al. showed that usage rates were higher among people with SMI compared to those without SMI during COVID-19 suggesting an increased utilization of remote methods of care delivery for SMI (37).

The Zentangle Method facilitates mindful practice by using visual art as the creative medium to bring attention to present experiences, offering a unique approach to integrate mindfulness and visual arts. Given the paucity of literature on the use of Zentangle as an effective form of therapy for individuals with SMI, we evaluated the feasibility and acceptability of a pilot 8-week Zentangle program delivered remotely in a group setting, as an adjunct treatment in this patient population. The study aimed to examine the potential therapeutic effects of the Zentangle program on comprehensive psychiatric symptoms within the SMI population, as well as to assess its broader influence on mindfulness, perceived stress levels, and overall quality of life. It was hypothesized that the Zentangle may lead to improvements in comprehensive psychiatric symptoms in addition to a favorable influence on mindfulness, perceived stress levels, and overall quality of life. Moreover, it was hypothesized that the maintenance effect would be observed post-intervention.

## 2 Materials and methods

### 2.1 Participants

Participants were recruited by word of mouth or physician referral between October 2020 and November 2020 in Central Massachusetts. The Zentangle program was publicized using flyers distributed at clubhouses providing psychiatric rehabilitation services, local hospitals, private practice clinics, and peer support groups. Inclusion criteria to participate in the program were as follows: (a) English-speaking; (b) adults between 18 and 65 years of age; (c) DSM-V diagnosis of schizophrenia spectrum disorder, major depression, or bipolar disorder; (d) psychiatrically and medically stable as determined by a board-certified psychiatrist; (e) access to basic technology to participate in the program. Participants were excluded if they were at imminent risk of suicide or injury to self or others. The study was approved by the Institutional Review Board at UMass Chan Medical School and written consent was obtained from the participants.

### 2.2 Zentangle program

The Zentangle program was conducted between January 2021 and April 2021 and included 8 weekly 90-min sessions. The sessions were led by two certified Zentangle instructors, each with over 15 years of practicing and instructing this specific art form. Beginner Zentangle kits were provided to all participants, which included white paper tiles, a black pen, graphite pencil, tortillon, and a Zentangle primer book, and were used throughout the program. During each session participants created their unique “tangles”—structured patterns and images on paper tiles—at their own pace. The Zentangle teachers used art as a direct and embodied tool to share mindfulness teachings with the group. Participants were encouraged to be non-judgmental and kind toward artistic creations and focus on each stroke leading to their individual art pieces. The first two sessions focused on the fundamental principles of the Zentangle Method (e.g., corner dots, border, string, tangle, shade). In sessions 3–7, participants were encouraged to create new designs with previously learned skills while reinforcing knowledge obtained from previous sessions. The Zentangle instructors emphasized and modeled core Zentangle teachings such as an emphasis on opportunities and de-emphasis on mistakes, positive reinforcement, and expression and trust in one's own ability to create. As the participants' skills and confidence grew, the level of difficulty and complexity rose.

### 2.3 Study protocol

This study protocol spans a total duration of 14 weeks, inclusive of a follow-up assessment occurring 5 weeks after the Zentangle intervention's conclusion. The study protocol was executed entirely in a virtual manner utilizing the HIPAA-compliant Zoom video platform. Data from the survey and measurement scales were acquired through individual virtual interviews conducted by study personnel. In week 1, a pre-intervention assessment is administered with the goal of obtaining baseline data from the measurement scales. Subsequently, the program unfolds over 8 weeks (weeks 2–9), featuring 90-min long weekly group sessions using the Zentangle method. One-week post-intervention (week 10), following the completion of the 8 Zentangle sessions, participants undergo reevaluation via measurement scales. Two weeks after the Zentangle program is finished (week 11), a virtual focus group meeting convenes to synthesize and debrief the Zentangle sessions, facilitated by a study team member. Five-weeks post-intervention (week 14), a final reassessment takes place using the same measurement scales. Additionally, a follow-up survey is administered with the specific aim of gathering data to assess the maintenance of the intervention's effects.

### 2.4 Measures

Participants completed the following measures over Zoom at baseline (1 week prior to the start of the program) and 1- and 2-weeks after the program: (1) The Brief Psychiatric Rating Scale (BPRS), which consists of 18 symptom areas to evaluate the impact of psychiatric symptoms such as depression, anxiety, and guilt on an individual's functionality of living. Graded on a scale from 1 (“not present”) to 7 (“extremely severe”) with an inter-rater reliability correlation coefficient of 0.67–0.88, the final score is composed of the sum of each individual item area where higher scores represent a higher overall burden of psychiatric symptoms (38, 39). (2) The Mindfulness Attention Awareness Scale (MAAS), which is a 15-item scale with Cronbach's alpha reliability (α) coefficient of 0.89, was used to assess the degree of mindfulness among participants. Questions were structured to describe an everyday life experience associated with a negative connotation and numbered from 1 (“almost never”) to 6 (“almost always”). The final score was obtained by calculating the mean performance of all 15 items with higher scores portraying more mindful behaviors (40). (3) The Perceived Stress Scale (PSS), which contains 10 items graded on a 4-point Likert scale (0 = never to 4 = very often) and α = 0.715. A higher total score indicates greater stress in everyday life (41, 42). (4) The Quality of Life Enjoyment and Satisfaction Questionnaire—Short Form (Q-LES-Q-SF), which includes 14 items with each item using a 5-point scale ranging from 1 (“very poor”) to 5 (“very good”) and an internal consistency reliability of 0.90. A total score is derived from 14 items with higher scores indicating greater life satisfaction and enjoyment (43, 44).

### 2.5 Focus group

Participants were invited to participate in a semi-structured, 30-min focus group over Zoom 1 week following completion of the 8-week program to better understand their experience with the Zentangle program. The following questions were used as a guide during the focus group session: what have the Zentangle sessions been like for you, what have you learned from Zentangle which you have applied to your everyday life, what changes in your mental health have you noticed while undergoing the program, and how would you define creativity. The focus group was conducted in English, facilitated by a research team member, and recorded and transcribed for analysis.

### 2.6 Data analysis

SPSS Staistics (version 27, IBM, Armonk, NY) was used to conduct quantitative data analysis. For each rating scale, one-way repeated measures analysis of variance (ANOVA) and paired *t*-tests were used to compare the scores across time points (baseline, 1-week post-intervention, and 5-week post-intervention). For qualitative data obtained from the focus group, the transcript was prepared verbatim and reviewed by two investigators (MS, VA) independently who were not involved in the focus group session or transcription process. The transcript was evaluated using thematic analysis (32, 45, 46). Dedoose software (Dedoose, Manhattan Beach, CA) was used to code the data extracts. Both evaluators read the transcript individually to familiarize themselves with the data and create initial codes. Themes for coded transcripts were developed through several iterations of analyzing the transcript. Upon establishing themes independently, the two evaluators met to finalize themes through discussion.

## 3 Results

Eight people with SMI enrolled in the Zentangle program. Six of them completed the 8-week program, two withdrew from the program due to technical challenges (internet access, familiarity, and comfort level of using the electronic device). Only participants who completed the entire program were included in the final data analysis. The participants consisted of one male and five females. The ages ranged from 21 to 62 years old (44 ± 17.7; mean ± SD). Three participants were diagnosed with major depressive disorder, two with schizophrenia, and one with bipolar disorder.

**Figure 1:**
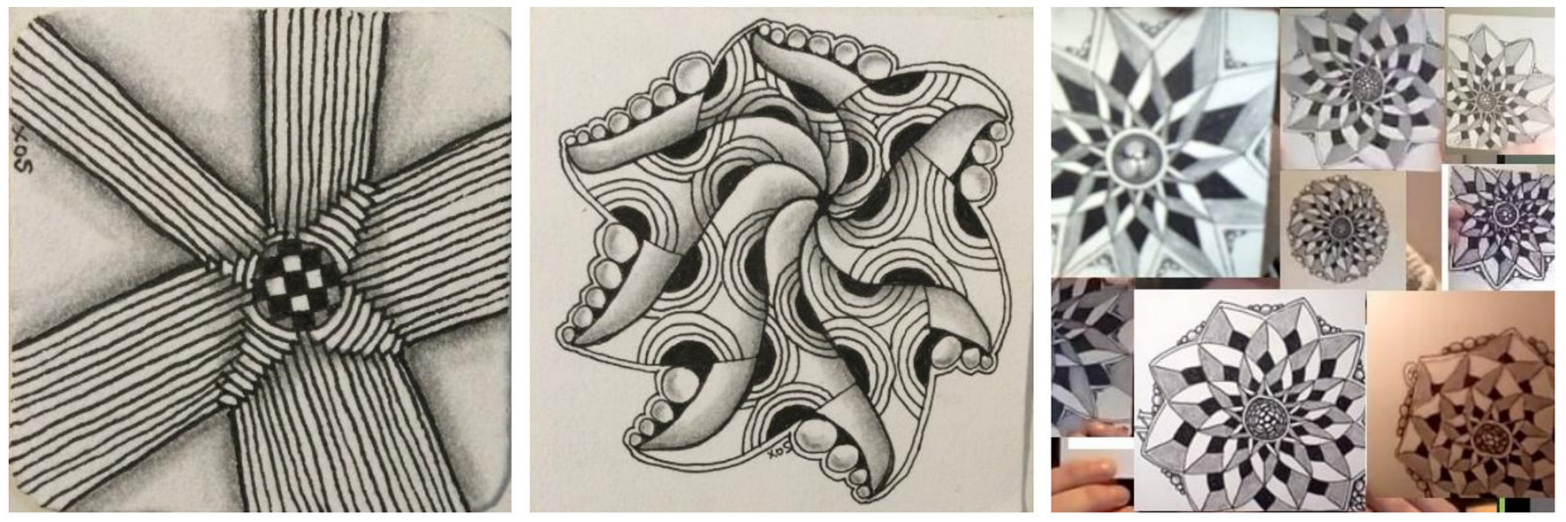
Examples of tangles created by participants in the Zentangle program.

Examples of tangles made by participants are shown in Figure 1.

One-way repeated measures ANOVA showed no significant difference across the three time points for any rating scale (data not shown, *p*'s > 0.05). However, there was a trend for a reduction in psychiatric symptoms in the BPRS total score across time points (*F*_2, 10_ = 3.614, *p* = 0.08). Pairwise comparisons showed a significant reduction in the BPRS total score between baseline and 5-week post-intervention (40.7 ± 9.1 vs. 33.7 ± 8.9, mean ± SD, *p* = 0.02) and a significant increase in the MAAS average score between 1- and 5-week post-intervention (3.5 ± 0.4 vs. 4.2 ± 0.7, mean ± SD, *p* = 0.04). Given the small sample size, corrections for the familywise error rate were not used for multiple comparisons. Qualitative thematic analysis of the focus group resulted in four themes (Table 1):

1) **Approaching mindfulness through Zentangle**. Participants described previous challenges they had with applying mindfulness in their lives and explained how Zentangle was a successful way for them to reach a state of awareness by practicing “just breathing, just relaxing and breathing”. Specifically, the act of “doing” art and focusing on the “stroke by stroke of a pencil” eased the way for participants to learn how to be present and reach perceived states of mindfulness in day-to-day life.
2) **Power of uncomplicated art creation**. Participants described the ease with which the Zentangle Method can be used in daily life. The ability to engage in art formation with simple tools like pencil and paper helped participants develop new perspectives on art formation. Moreover, participants identified the value of routine applied to artmaking as a positive trait of Zentangle. However, one participant shared the following: “it was really hard for me at first to do Zentangle a lot because I'm doing this and it doesn't have a bigger purpose”, suggesting that the lack of organized direction when tangling can be interpreted as a challenge by some people.
3) **Understanding the value of self-appreciation**. Participants explained their struggles with self-criticism and judgment that overwhelmed their daily living. A general understanding of how SMI can negatively impact one's ability to reach normal states of “gratefulness” was shared. Creating a judgment-free environment by being part of the Zentangle program brought about a sense of self-appreciation and positive energy according to participants.
4) **Fostering a positive environment during the COVID-19 pandemic**. Participants expressed their frustration with the COVID-19 pandemic and how they felt relieved by participating in the Zentangle program. Practicing art in a group setting helped alleviate feelings of loneliness and isolation that were common during the pandemic. Moreover, participants shared how they “felt happier” attending sessions each week and that the structured schedule of the program was “really fun”.

**Table 1:** Themes and excerpts from the focus group participants.

**Theme**	**Excerpt**
Approaching mindfulness with Zentangle	“Well, all of my doctors are always trying to get me to learn mindfulness meditation and it makes me mad. Makes me more anxious and I hate it. But I mean this is (Zentangle) really the same thing. Just you're actually drawing while you're doing (mindfulness). You're not just sitting, having to watch your thoughts like which is horrible to me.” “They (instructors) try to teach it to work, stroke by stroke and, you know, not to think about what ahead, what you're doing.” “I think practicing focusing on what I'm doing at that second, you know, and not trying to look ahead.”
Power of uncomplicated art creation	“Yeah, it (Zentangle) teaches you that you don't need, you know as you say your whole art supply to be able to create something satisfying, you know.” “I haven't stopped. It's like the only thing I want to do anywhere.”
Understanding the value of self-appreciation	“Self-criticism, it's all the rest of the time, so doing this (Zentangle) is like the one time when I am not hearing critical voices constantly in the head.” “With depression, you can't feel grateful. You can't, it's faking it if you try to do that. But this one (Zentangle) feels a little bit like easier, because you're actually like feeling gratitude in that minute for what you're doing.”
Fostering a positive environment during the COVID-19 pandemic	“Because of the COVID. I mean, we're all been stuck inside and it's just been such a pleasure to have something (Zentangle) that you know you do every week.” “Yeah, yeah, and I had accomplished something when I haven't accomplished anything in like a year.”

Based on the data from the follow-up survey, four of the six participants continued practicing Zentangle 5 weeks after completing the program. Participants noted that the repetitive, non-judgmental nature of tangling was “relaxing” and “meditative”. In addition, they considered Zentangle as a “satisfying” activity, particularly in the context of the pandemic.

## 4 Discussion

Research on the Zentangle Method remains relatively scarce, with a particular shortage of studies focusing on individuals with SMI. The aim of our investigation was to contribute to this underexplored scientific domain by conducting an assessment of the feasibility and acceptability of the virtually delivered Zentangle Method within the context of individuals living with SMI. In this pilot study, we adopted a comprehensive mixed-methods approach, enhancing the study by incorporating both quantitative measurements and qualitative insights derived from focus group analysis. One advantage of the study is the use of diverse set of validated outcome measures, including the BPRS, MAAS, PSS, and Q-LES-Q-SF, to assess psychiatric symptoms, mindfulness, stress, and quality of life. This comprehensive approach provides a holistic view of participants' mental wellbeing. Our preliminary quantitative data suggest that the use of Zentangle for participants with SMI may have a positive impact on overall psychiatric symptoms and mindfulness. These results align with the research outcomes reported by Chen et al., which indicated a positive correlation between improved mental wellbeing and individuals with psychosis in Taiwan. In addition, a therapeutic effect on mindful attention was achieved 5 weeks after completion of the 8-week program, suggesting that a longer-term Zentangle program might be more beneficial for future participants (47). Cognitive impairment is considered a fundamental feature of SMI and is linked to increased disability, poor health, and suboptimal recovery outcomes (48, 49). Impairment in cognitive domains like working memory, attention, and processing speed are detectable early in the course of illness with attention being a primary cognitive deficit (50). Mindfulness practices may bolster attention-related behavioral responses by modifying subsystems of attention (51).

While Randomized Controlled Trials (RCTs) have been instrumental in evaluating the effectiveness of Zentangle intervention, together with the work from Cheung et al., our study demonstrates the added value of employing a mixed-method approach. This approach offers a more holistic and nuanced understanding of the Zentangle program's effects, capturing the intricacies of participant experiences and outcomes beyond what RCTs alone can provide. The qualitative data gathered from the focus group interview corroborates evidence for enhancements in the capacity to attain states of mindfulness among participants. Mindfulness interventions are intended to ease distress by changing how people relate to difficult inner experiences. Over the years, the integration of mindful practices into cognitive and other psychoeducational interventions has led to meaningful benefits across a range of psychological disorders (20). Individuals living with SMI face persistent and debilitating psychosocial stressors such as social rejection, stigma, and subjective distress. Internalization of these experiences can have a detrimental impact on daily living with the potential to negatively impact the ability to reach mindful states and improve their attention ability (52). Participants in our pilot study identified personal struggles with practicing mindfulness in the past. The Zentangle Method allowed participants to actively participate in the creation of art as they worked toward reaching states of mindfulness. Through practicing the Zentangle Method, participants experienced dispositional mindfulness, described as open or receptive awareness of and attention to what is taking place in the present. As a mindfulness-based art intervention, Zentangle is grounded on mindful teachings within the structure of a creative art framework and has the potential to provide therapeutic benefits to people with SMI (27, 28). Moreover, through the focus group analysis, the “power of uncomplicated art creation” was isolated as a theme, emphasizing the ease and flexibility of Zentangle practice. This distinguishes the Zentangle Method from other mindfulness-based art therapies, which are typically resource-intensive, time-consuming, and require months to a year for observable effects (24).

Implementing Zentangle in a group format allowed participants to build a virtual community and share their artwork. This reduced a sense of social isolation, which has been commonly experienced during the pandemic. Another potential strength of our study is its complete virtual format, which enables participants to engage in the program, effectively eliminating transportation and location constraints. This approach significantly increases accessibility, extending the program's potential impact to a wider audience (37, 53). In addition, the Zentangle Method was designed in a flexible and accepting manner and promoted positive feelings such as gratitude and self-accomplishment to help participants overcome negative feelings of self-criticism and failure. These results align with the findings of recent studies investigating Zentangle, which have consistently indicated notable enhancements in self-compassion and positive affect (33, 34, 54). This suggests that engaging in Zentangle fosters a sense of self-compassion, allowing individuals to be more understanding and kind to themselves, while also promoting an overall positive emotional state. Notably, 67% of participants continued to practice Zentangle 5 weeks after the program was over, highlighting the acceptability of the Zentangle Method in the SMI population.

Our pilot study has several limitations. First, the small sample size diminished the power of analysis. Second, most participants were female as has been the case in many arts-based programs (55, 56); the feasibility and generalizability of Zentangle in male participants with SMI remains unclear. Third, there was a lack of control group which makes it difficult to attribute these findings to the intervention itself among other factors such as engaging in a structured program and gathering socially. Additionally, two enrolled participants did not complete the program due to challenges with technology. Digital intervention delivery may pose a barrier to individuals with limited experience utilizing services accessed online. The virtual format, with its accessibility benefits mentioned above, also underscores the importance of addressing the digital divide and ensuring individuals with limited online experience can effectively engage in these interventions. Future studies may examine the effect of Zentangle compared with other mindfulness-based programs for those living with SMI with a focus on larger scale, more rigorous methodology to uncover the specific benefits gained from such programs. Combining pharmacologic with psychosocial interventions like Zentangle may have the potential to augment life satisfaction, social engagement, and treatment of mental illness.

Preliminary findings from this pilot study demonstrated the feasibility and acceptability of the Zentangle Method and provided preliminary evidence of its positive impact on general psychiatric symptoms and mindfulness. Participants found the Zentangle Method of drawing, in which attentive states are sought while actively participating in the creation of art, to be beneficial in their efforts to achieve states of mindfulness. Moreover, participants were able to create a virtual community and share their artwork using Zentangle in a communal setting which helped to alleviate feelings of social isolation often experienced during the pandemic. These findings support further exploration of Zentangle as an adjunct art intervention for people with SMI.

## Data availability statement

The raw data supporting the conclusions of this article will be made available by the authors, without undue reservation.

## Ethics statement

The studies involving humans were approved by University of Massachusetts Chan Medical School IRB. The studies were conducted in accordance with the local legislation and institutional requirements. The participants provided their written informed consent to participate in this study.

## Author contributions

MS: Conceptualization, Data curation, Formal analysis, Investigation, Project administration, Validation, Visualization, Writing – original draft, Writing – review & editing. AC: Conceptualization, Data curation, Formal analysis, Investigation, Validation, Visualization, Writing – original draft, Writing – review & editing, Methodology. VA: Data curation, Formal analysis, Investigation, Methodology, Writing – original draft, Project administration. XF: Data curation, Formal analysis, Investigation, Methodology, Project administration, Conceptualization, Funding acquisition, Resources, Supervision, Validation, Visualization, Writing – original draft, Writing – review & editing.
